# Commentary: Wild psychometrics: Evidence for ‘general’ cognitive performance in wild New Zealand robins, *Petroica longipes*

**DOI:** 10.3389/fpsyg.2017.00165

**Published:** 2017-02-08

**Authors:** Paul M. W. Hackett

**Affiliations:** ^1^Department of Psychology, University of GloucestershireCheltenham, UK; ^2^Department of Psychology, University of CambridgeCambridge, UK; ^3^School of Communication, Emerson CollegeBoston, MA, USA

**Keywords:** facet theory, cognition, avian intelligence, comparative psychology, “g” factor in intelligence, psychometrics

Studies into the psychometric structure of human intelligence and cognitive ability have identified a “*g”* or *general* factor, e.g., Buckhalt (2002), and in comparative psychology, in primates: Reader et al. (2011) and in rodents: Matzel et al. (2003). As well as this “*g”* factor, intelligence is conceptualized as possessing multiple specific intelligences. Due perhaps to more recent discoveries regarding the complexity of birds cognitive abilities (see, Emery, 2016), birds have become the subject of psychometric investigation (e.g., Ackerman, 2016).

Seminally, Shaw et al. (2015) assembled a test battery for avian cognition to assess the cognitive abilities in New Zealand robins (*Petroica longipes*). Their test battery was comprised of a series of, “tasks based on established measures of avian cognitive performance: a motor task, color and shape discrimination, reversal learning, spatial memory and inhibitory control.” (Shaw et al., 2015, p. 101). These scientists found robins to vary greatly in their ability to solve tasks. They also discovered weak, positive, non-significant correlations between performances on most tasks. They analyzed test performance using principle component analysis (PCA) using the criteria for extracting components as being the possession of an eigenvalue of unity or above. All sub-tests were found to load positively on the first component, which explained >34% of between-test variance. As a consequence of this finding, the authors identified this first factor aa a “*g”* factor, and concluded that New Zealand robins, tested in their wild habitat, displayed a general cognitive factor analogous to the human “*g”* factor. They continue to draw similarities between their own research and the body of literature on human general intelligence. In their article, Shaw et al. (2015) state that: “In human psychometric testing, individuals' scores in tests of diverse cognitive processes are positively correlated, and a “*g”* factor typically accounts for at least 40% of total variance” (Shaw et al., 2015, p 101).

However, several researchers, (e.g., Guttman, 1965, 1981; Koop, 1985; Guttman and Levy, 1991) have expressed concerns with the use of PCA to analyze tests of cognition as this procedure embodies specific assumptions regarding the data being analyzed that may not be met. For example, PCA requires that the data being subjected to PCA possesses the following characteristics: that the data is parametric in nature; that there is a correspondence between the data and the psychological structure of the construct being assessed; that comparability exists between the measurement scales in different sub-tests, etc.

Within human psychometrics questions have been asked regarding the nature of the relationship between general and specific forms of intelligence. Rimoldi (1951) hypothesized upon the nature of a “*g”* factor in intelligence. Koop (1985) notes how Rimoldi proposed that the “*g”* component was better understood as being a second-order factor (as being a product of specific intelligences) rather than as a primary factor with secondary specific intelligences. A psychometric approach that has been employed to analyse human cognition (and many other forms of behavior and experience) is that of facet theory (see Hackett, 2014, 2016). Typically, when a facet theory perspective is utilized, a definitional framework, known as a mapping sentence, is developed and research instruments are designed to investigate this initial propositional definition. Data reduction techniques (similar to PCA, but having different assumptions about the data), such as smallest space analysis (SSA) and partial order scalogram analysis (POSA) are used to test the veracity of the mapping sentence. Another way in which facet theory has been used is to analyse existing data sets. The data set produced through Shaw et al.'s research into New Zealand robin's cognitive performance is a data set that would be suited to such analysis.

An example of how a facet theory approach has been sued to interrogate an existing data set is Koop's (1985) reanalysis of Rimoldi's (1951) data using facet theory's analytic procedure: SSA and found a radial (qualitative) array of sub-tests (geometrical; numerical and verbal tests). Furthermore, he discovered that each of these sub-types of intelligence sub-tests were modified in terms of whether a specific sub-test required: inference, application or learning. Other researchers have divided the “*g”* intelligence into two forms: fluid (gf) and crystalized (gc) general intelligence (Cattell, 1963; Horn, 1988). Beauducel et al. (2001) used the facet theory approach to investigate the structure of fluid and crystalized general intelligence. They discovered support for the discrete existence of the tests of: verbal, numerical and figural abilities, with these being divided into: fluid (gf) and crystalized (gc) general intelligence, in a 2 × 3 structure.

Shaw et al.'s (2015) psychometric test battery and framework has demonstrated cognitive structures analogous to human intelligence and their findings are an extremely important steps toward better understanding how birds cognitively experience and process their world. Of particular relevance to this commentary is the fact that Shaw and colleagues identification of a complex structure to exist within avian intelligence offers a new research area for facet theory.

To conclude, facet theory approach has allowed the depiction and modeling of human cognitive processes and for understanding human intelligence. It is the contention of this author that facet theory provides a way to conceptualize and design avian cognition research. The approach also provides a way to analyse data that arises from research designed in this way using non-parametric techniques (e.g., SSA and POSA) to reveal the structure of avian intelligence in the form of a mapping sentence. By using a mapping sentence to design research and non-parametric statistical analyses this allows the investigation of the structure of avian intelligence in a way that does not make unsubstantiated assumptions of the psychometric qualities of the data. Furthermore, facet theory based research produces cumulative and directly comparable results across studies employing a faceted design. Thus, the use of facet theory could potentially develop greater understanding of avian cognitive processes.

## Author contributions

The author confirms being the sole contributor of this work and approved it for publication.

### Conflict of interest statement

The author declares that the research was conducted in the absence of any commercial or financial relationships that could be construed as a potential conflict of interest.
